# 

**Published:** 1885-03-20

**Authors:** 


### EDITORIALS AND MISCELLANEOUS.

☞ **Notice.**--- Subscribers in arrears are requested to remit their dues at once. Please take due notice and govern yourselves accordingly.

### EDITORIAL NOTICES.

THE Texas State Medical Association meets at Houston on Tuesday, the 21st of April next.

MEDICAL SOCIETIES —The secretaries of the State Medical Societies are invited to report the times of meeting in this Jonrnal.

THE Louisiana State Medical Society is appointed to meet at New Orleans April 21st, 1885.

QUININE ADULTERATED.—We are cautioned in regard to the adulteration of quinine, which is said now to prevail to an unusual extent. Many samples now on the market do not contain fifty per cent. of the pure drug.

THE Georgia Medical Society will meet the present year at Savannah, on the 15th of April next. A large and interesting meeting is anticipated. Let all attend who possibly can do so.

GEN. GRANT'S HEALTH.—The papers report Gen. Grant as declining in health, and that he cannot live long. He has some affection of the tongue and throat, supposed to be cancerous, and yet there is a vagueness and indefiniteness in the statements of his physicians that leaves us in doubt as to his actual condition.

PRIZE OFFERED.—There is to be a handsome prize ($900) awarded by the Royal Academv of Turin – the Capital of Sardinia, Italy—to the author of the best work that is issued between 1883 and 1886 on any subject connected with physics, chemistry, physiology, geology, geograpny or statistics, or to the author of any important discovery. The prize is open to all countries.

SURGICAL INSTRUMENTS, ETC.—We invite attention to the advertisement of Isaac Phillips, agent for surgical instruments; orthopædic appliances, drug cases, speculums, saddle-bags, &c., &c.—everything in the doctor's line. Mr. Phillips is a polite, energetic and prompt business man. In writing to him, please mention this Journal.

MELLIN'S FOOD.—This preparation is regarded as a superior food for infants and invalids. It contains all the essential elements for nutrition in a form which accords with the laws of physiology. See the advertisement of this article in our Journal, by that excellent house of Doliber, Goodale & Co., of Boston.

INFLUENZA.—There is prevailing in this section (Atlanta, Ga.) a form of catarrhal fever of peculiar type, which is characterized in most cases by chilly sensations in its commencement and febrile reaction in the afternoon, aching in the limbs and back, etc., indicating malarial influence. The ordinary cough remedies exert but little influence upon the symptoms. Calomel, followed by quinine and Dover's powder, give the best results in treatment.

MENINGITIS.—A number of cases of cerebro-spinal meningitis have occurred in Atlanta during the last month. The prevalence of influenza has been mentioned. It has been observed that cerebro-spinal meningitis is very liable to occur during the prevalence of epidemic influenza, which seems to indicate some sort of relationship between the two affections.

WRITE ! WRITE !—Our friends are requested to write for our Journal. We want short, pointed and practical articles. Reports of cases, containing results of treatment, whether successful or otherwise, are desired. Formula which are new, and have proven efficient in practice, will be appreciated and duly credited to the authors. Brethren, publish what you know, and thus advance the progress of medicine and do good to your fellow men.

MESSRS. SHARPE & DOHME'S DRUG CASE.—The beautiful pocket drug case presented as a prize in physiology at the recent brilliant commencement exercises of the Southern Medical College, Atlanta, Ga., was from that splendid house known as SHARPE & DOHME, Manufacturing Chemists and Pharmacists, Baltimore, Md. It contained twenty-five phials, filled with a variety of beautiful sugar-coated and gelatine-coated pills. We can recommend this case as not only beautiful in appearance and convenient in its arrangement, but as containing pure drugs and prepared by a business house of a high order and widely known throughout the whole country as an active, enterprising and reliable establishment.

They run an advertisement on the second cover page of this Journal.

### *RECEIPTED.*

1884.—Drs J E Pope, Jno Hardeman, J T Suggs to June, Wm Tate, Isaac Yates, Sam'l Bomar, James Sylar, J D Bayley, E L Morton, Simon Chandler, L B Camp, Tho A Morgan, Jackson Billups.

1885.—J A Smith, A M Duncan, D O McGehee, H T Webb, Wm Childs, T L Turk, T B Meacham, Wm Mayson, T T Christian, James Lawton, Elias Miller.

